# Resolutions of Respect—Massachusetts Board of Registration in Dentistry

**Published:** 1898-03

**Authors:** 


### Obituary.

### RESOLUTIONS OF RESPECT—MASSACHUSETTS BOARD OF REGISTRATION IN DENTISTRY.

Whereas, In the sudden death of Dr. E. V. McLeod, which occurred November 24, 1897, the Massachusetts Board of Registration in Dentistry has lost one of its original members, who during its existence of eleven years has served as secretary.

*Resolved*, That in the death of Dr. McLeod the Board has sustained the loss of one who has been most efficient and faithful to its duties, giving an immense amount of his time to its work.

*Resolved*, That as an examiner he displayed wisdom and discretion and wonderful tact in dealing with men.

*Resolved*, That in his contact with his associates on the Board his kindliness of nature and generosity of heart will always be remembered with the warmest affection.

*Resolved*, That we extend our sincere and heartfelt sympathy to his bereaved widow in this her great affliction.

*Resolved*, That these resolutions be entered on the records and a copy be sent the widow of the deceased and to the several dental journals for publication.

(Signed) JOHN F. DOWSLEY.
GEORGE E. MITCHELL.
THOMAS J. BARRETT.
DWIGHT M. CLAPP.

